# Home and health in people ageing with Parkinson’s disease: study protocol for a prospective longitudinal cohort survey study

**DOI:** 10.1186/1471-2377-13-142

**Published:** 2013-10-09

**Authors:** Maria H Nilsson, Susanne Iwarsson

**Affiliations:** 1 Department of Health Sciences, Lund University, Box 157, SE-221 00 Lund, Sweden

**Keywords:** Activities of daily living, Falls, Housing, P-E fit, Walking

## Abstract

**Background:**

With an increased life expectancy for the general population as well as for those ageing with chronic diseases, there are major challenges to the affected individuals and their families, but also to health care and societal planning. Most important, an increasing proportion of older people remain living in their ordinary homes despite health decline and disability. However, little is known about the home and health situation of people ageing with Parkinson’s disease (PD), and older people are often excluded from PD-research.

**Methods/design:**

The overall aim of the present project is to generate knowledge on home and health dynamics in people with PD, with an explicit attention to PD-specific symptomatology. We will concentrate on aspects of home and health captured by state-of-the-art methodology from gerontology as well as PD-research, health science and rehabilitation. This study protocol describes a longitudinal cohort survey study that includes a baseline data collection and a 3-year follow-up. Both data collection waves include self-administered questionnaires, structured interviews, clinical assessments and observations during home visits effectuated by research staff with project-specific training. In order to arrive at a follow-up sample of N=160, 250 participants identified by PD specialist nurses are being recruited from three hospitals in southern Sweden. With no lower or upper age limit, only those diagnosed with PD since at least one year were included. The exclusion criteria were: difficulties in understanding or speaking Swedish and/or cognitive difficulties/other reasons making the individual unable to give informed consent or to take part in the majority of the data collection. The data collection targets environmental factors such as assistive devices, social support, physical environmental barriers, accessibility problems and perceived aspects of home. A broad variety of instruments tap PD-specific problems (e.g. freezing of gait, fear of falling) and health-related issues such as general self-efficacy, body functions, activities and participation.

**Discussion:**

This project will produce knowledge to the benefit of the development of health care and societal planning that targets people ageing with PD, ultimately promoting activity and participation and an increase of the number of healthy life years for this sub-group of the population.

## Background

With an increased life expectancy for the general population as well as for those ageing with chronic diseases, there are major challenges to the affected individuals and their families, but also to health care and societal planning. Most important, an increasing proportion of older people remain living in their ordinary homes despite health decline and disability. Since the existing knowledge on the complexities of home and health along the process of ageing is based solely on general population samples, there is an urgent need to also study sub-groups with specific diagnoses.

Parkinson’s disease (PD) is one of the most common chronic neurodegenerative disorders, and the symptoms deteriorate over time, with increasing complexity. The average age at onset is 60 years, and people live for about 20 years with the disease. People with PD are more likely to move to assisted living facilities and at an earlier age [1], and falls are among the leading reasons for nursing home admittance [2]. This causes high costs to society [1] and large consequences for those affected. Despite this, PD-studies that have systematically examined home and health dynamics are lacking, and older people are often excluded from PD-research [3]. Consequently, there are knowledge gaps about the life situation for those ageing with PD, with a limited understanding concerning home and health dynamics in this sub-group of the population.

Although PD is characterized by four cardinal motor symptoms (i.e. tremor, bradykinesia, rigidity and postural instability [4]), also non-motor symptoms are common, e.g. cognitive problems, depression and fatigue [5]. Difficulties in performing activities of daily living are present even at the early stage of PD [6], and about 75% have gait and balance problems [7,8]. People with PD have an increased risk of falling (including experiencing near falls) than others of the same age, and a fear of falling is also more pronounced and common [9-11]. Most of the falls occur indoors at home while walking or turning [12]. People with PD also have disease-specific activity limitations caused by freezing of gait (FOG). FOG is associated with certain activities (e.g. turning) and environmental factors (e.g. being in a confined space), and it most commonly occurs in the home environment [13]. Still, the knowledge on person-environment interactions among people ageing with PD and how such dynamics interact with the PD symptomatology is almost non-existing. Due to the negative consequences of PD on everyday life, PD-specific symptoms and problems need specific attention. This in particular since dopaminergic treatment strategies insufficiently tackle balance problems and people with PD also develop non-dopaminergic symptoms (e.g. falls, dementia). In order to increase the number of healthy life years despite the consequences of ageing with a chronic disease, efficient rehabilitation is of the utmost importance [14]. There is, however, limited evidence for the efficacy of fall prevention [15], housing adaptations and mobility device provision in people with PD. Moreover, even less is known about what is needed to support the development of adequate housing options and other kinds of societal support for people ageing with PD.

Turning to the conceptual and theoretical perspectives underpinning the present project, they represent a fusion of frameworks applied mainly in gerontology, health science and rehabilitation. In the ecological theory of ageing (ETA) [16,17], the person is defined as a set of competencies and the environment is defined in terms of its demands (environmental press). When health declines the environmental pressure often exceeds the individual capacities, resulting in person-environment fit (P-E fit) problems and negative health outcomes. The International Classification of Functioning, Disability and Health (ICF) (WHO, 2001 [18]) is commonly used as a conceptual framework to define and describe health and health-related outcomes. The main components of ICF are body function and structure, activity and participation, interacting with environmental and personal factors. According to the ICF, environmental factors do not only include the built and natural environment and assistive technologies (e.g. assistive devices [ADs]), but also factors such as support by others. To grasp the impact of disease, explicit knowledge is needed on how the interactions between a specific state of health and environmental factors affect the individual’s daily life. In a recent WHO and World bank report, the importance of focusing on accessibility issues and environmental barriers in order to promote activity and participation was put forward [19]. Until now, PD-research has not focused on such issues.

Since no previous PD-studies have combined these perspectives, a set of preparatory and explorative studies were conducted as part of the planning process for the present project. Using data from an existing cross-national database [20], the results suggest that very old people with self-reported PD (n=20) live in dwellings with more accessibility problems, perceive their home to be “less usable in relation to activities” and have significantly more unmet needs concerning assistive devices for personal care and protection, e.g. related to shower/bath, toileting [21,22] than matched controls. That is, the results suggest that very old people with PD need specific attention regarding aspects of home, functional limitations and ADs. However, in order to generate valid results that can be generalized to people ageing with PD, longitudinal projects that involve participants with a confirmed PD diagnose are imperative.

The overall aim of the present project is to generate knowledge on home and health dynamics in people with PD, with an explicit attention to PD-specific symptomatology. In order to determine key issues of importance for the development of interventions that efficiently target the life situation of people ageing with PD, we will concentrate on aspects of home and health captured by state-of-the-art methodology from gerontology as well as PD-research, health science and rehabilitation. Examples of specific aims are:

● To explore the interactions between perceived and objective aspects of home

● To determine which functional limitations and physical environmental barriers that contribute the most to housing accessibility problems, and whether the contributing factors differ among fallers versus non fallers.

● To identify which physical environmental barriers that induce the most accessibility problems among those with FOG and/or a fear of falling.

● To define PD-specific aspects (motor and non-motor symptoms) that may hamper perceived usability of the home in relation to activity performance.

● To identify the use and unmet needs for ADs and such changes over time

● To determine how perceived and objective aspects of home are related to activity limitations and participation restrictions, and how such interactions evolve over time.

## Methods/design

### Research design

This longitudinal cohort survey study includes baseline assessments and a 3-year follow-up. Both data collection waves include state-of-the-art self-administered questionnaires, structured interviews, clinical assessments and observations during home visits, targeting a wide range of aspects of home and health.

### Participants and recruitment

#### Inclusion and exclusion criteria

Only those with a PD diagnosis (G20.9 according to the ICD-10) were included. Since it might change [6], the participants should have had their PD-diagnosis for at least one year prior to inclusion. The exclusion criteria were: difficulties in understanding or speaking Swedish, and/or cognitive difficulties/other reasons making the individual unable to give informed consent or take part in the majority of the data collection.

#### Estimation of statistical power

Our estimates of the statistical power are based on data available in the existing ENABLE-AGE database including longitudinal data from 1,918 very old people in five European countries (PI: S. Iwarsson) [20]. A variable on the perceived aspect of home labeled “Meaning of Home” constituted the primary variable for the power and sample size estimates. The ENABLE-AGE data indicated that the longitudinal difference is normally distributed, with a standard deviation of 1.04. If the true difference with reference to average responses is 0.23, we will need to study 162 individuals in order to reject the null hypothesis that this difference is 0 with 80% probability (power). The probability for a type I error is 5%. Based on previous experiences, a dropout rate of 35% is anticipated over a 3-year period, and we will therefore include 250 individuals.

#### Recruitment of participants

The participants were recruited from three hospitals in Region Skåne, Sweden, selected by PD specialist nurses. For the first phase, participants were recruited from the local hospitals in Kristianstad and Hässleholm. All those registered with a PD diagnosis at the departments responsible for PD-care were considered eligible for inclusion. Since the number of participants available from the two local hospitals did not correspond to the required sample size (see below), in the second phase additional participants were recruited from Skåne University Hospital. All those diagnosed with PD that visited the Department of Neurology in Lund as outpatients during 2012 were identified, leaving us with a target sample considered sufficiently large (data collection is ongoing; n=223 completed).

### Ethical approval

The study was approved by the Regional Ethical Review Board in Lund, Sweden (No. 2012/558), and written informed consent is obtained at the home visit.

### Procedures

The baseline data collection (completed during 2013) is administered and performed by two project administrators (experienced reg. occupational therapists) that underwent project-specific training. They send out written information about the study (including the invitation to participate) by post to potential participants, who are subsequently also telephoned and offered the possibility to pose complementary questions about the study. With those that accept to participate and for practical reasons, some basic descriptive information is gathered by telephone, such as when the individual usually is feeling at best during the day; self-rated PD-severity (mild/moderate/severe); comorbidities; advanced PD-treatment, and work situation. The project administrators schedule individual home visits for a time of day at which the participant in question stated that he/she usually feels at best.

In order not to burden the participants more than necessary during the home visit, the participants receive the selection of self-administered questionnaires in the post ten days in advance of the home visit (Table 1). At the home visit, the project administrator scrutinizes the questionnaires, e.g. checks for missing data. During the home visit a structured interview is then combined with clinical assessments and an observation of the home environment. In cases where the participant experiences the data collection as too strenuous, he/she is offered the possibility to stop the data collection and continue on another day. In such cases a second home visit is scheduled within a maximum of 14 days. If exceeding this time limit, the participant is offered a renewed baseline assessment.

**Table 1:** Self-administered questionnaires sent out before the home visit

**Variable**	**Questionnaire**	**Number of items**	**Number of response categories (scoring)**	**Total score range (interpretation)**	**Time reference when rating**
Activities in Daily Living (ADL)	PADLS	1	5 (1–5)	1-5 (higher = worse)	last month
Walking difficulties in daily life	Walk-12G	12	3 (0–2): items 1–3 5 (0–4): items 4-12	0-42 (higher = worse)	last two weeks
Freezing of gait	FOG-Qsa	6	5 (0–4)	0-24 (higher = worse)	last week (except item 3)
Fall-related self-efficacy	FES-I	16	4 (1–4)	16-64 (higher = worse)	-
Activity avoidance due to a risk of falling	mSAFFE	17	3 (1–3)	17-51 (higher = worse)	-
General self-efficacy	GSE	10	4 (1–4)	10-40 (higher = better)*	-
Non-motor symptoms	NMSQuest	30	3: Yes/Don’t know/No	Not applicable	last month
Fatigue	NHP-EN	3	2: Yes/No	0-100 (higher = worse)**	at present

*A mean score can be used as an alternative to using a summed total score.

**Alternatively, those that respond yes to at least one item are considered to have fatigue.

*Abbreviations*: *FES-I* Falls Efficacy Scale-International, *FOG-Qsa* self-administered version of the Freezing of Gait Questionnaire.

*GSE* General Self-Efficacy Scale, *NHP-EN* the Energy section of the Nottingham Health Profile (items number 1, 12 and 26 in NHP).

*NMSQuest* The Nonmotor Symptoms Questionnaire (this screening renders no total score), *mSAFFE* = modified Survey of Activities and Fear of Falling in the Elderly,

*PADLS* Parkinson’s Disease Activities of Daily Living Scale, *Walk-12G* the generic Walk-12.

At the 3-year follow-up (planned for 2016), data collection will be conducted by a PhD-student and a project administrator with academic degrees as health care professionals. Both will undergo the same training as the baseline data collectors.

### Data collection instruments

#### Measurement properties

The self-administered questionnaires (see Table 1) have shown satisfactory measurement properties (including validity and reliability) for this administration mode in PD-research [23-28]. Based on studies targeting the older population in general [20,29-31], satisfactory measurement properties apply also for the instruments administered at the home visit. Variables at a glance are presented in Table 2.

**Table 2:** Variables at a glance in relation to conceptual and theoretical perspectives

**ICF (WHO, 2001)**	
	• Personal factors: e.g. sex, age, coping, self-efficacy
	• Body functions: e.g. motor and cognitive symptoms, pain
	• Activity: e.g. activities of daily living (ADL), walking
	• Participation: e.g. recreation and leisure, social interactions
	• Environmental factors: e.g. physical environmental barriers, assistive products and technology, support by others, and relationships
**P-E fit and perceived aspects of home**	
	• Personal component (P): functional limitations and dependence on mobility devices
	• Environmental component (E): physical environmental barriers
	• Perceived aspects of home: housing satisfaction, usability of the home, meaning of home and housing-related control beliefs
**PD-specific aspects/problems**	
	• Motor symptoms: tremor, brady- and hypokinesia
	• Non-motor symptoms: e.g. depression and fatigue
	• Gait: e.g. freezing of gait (FOG)
	• Balance problems: e.g. near falls, falls, fall-related self-efficacy, activity avoidance due to the risk of falling, dual task difficulties, and a clinical assessment of the postural response in relation to an external perturbation
	• Complications of therapy: e.g. experiencing fluctuations and/or dyskinesias
Since people with PD suffer from slowness and difficulties in performing daily transfers, two timed tests are included that tap mobility: walking and rising from a chair.	

*Abbreviations*: ICF = the International Classification of Functioning, Disability and Health, P-E fit = Person-Environment fit, PD = Parkinson’s disease, WHO = World Health Organization.

#### Descriptive variables

Descriptive variables capture age, sex, PD-duration and severity (clinician-rated and self-rated), disease subtype (tremor-dominant or Postural Instability and Gait Difficulty [PIGD]–dominant PD [32]), comorbidities, living situation, education, work situation, type of housing, etc.

#### Environmental aspects

##### Objective aspects of home

Based on the notion of P-E fit [16], objective aspects of the home are operationalized as the number and type of physical environmental barriers in the home and the exterior surroundings, and the magnitude of accessibility problems, according to the Housing Enabler (HE) [33]. With this instrument [33,34] administered in three steps, physical environmental barriers are objectively assessed based on national standards for housing design and juxtaposed with the individual profile of functional limitations:

Step 1) Interview and observation of functional limitations (12 items) and dependence on mobility devices (2 items): difficulty in interpreting information; visual impairment; blindness; loss of hearing; poor balance; incoordination; limitations of stamina; difficulties in moving head; reduced upper extremity function; reduced fine motor skill; loss of upper extremity skills; reduced spine and/or lower extremity function, and dependence on walking aids/wheelchair. All items are dichotomously assessed (Present/Not present). Step 1 renders a profile of functional limitations and provides a sum score of the number of functional limitations (range = 0–12). The data collected under Step 1 of the HE can also be used as a health aspect variable.

Step 2) Observation and dichotomous assessment (Present/Not present) of 161 physical environmental barriers indoors in the home (n=87), at entrances (n=46) and in the immediate exterior surroundings (n=28). Environmental barriers are objectively judged in relation to the applicable national standards for housing design. This step does not require any involvement of the participant. Step 2 generates the sum score variable ‘number of environmental barriers’ (range 0–161, or divided into the sub-domains indoors, entrances, exterior surroundings) and also provides a detailed account of the type of environmental barriers present.

Step 3) Based on the results of steps 1 and 2, the extent and character of the accessibility problems are calculated by means of instrument-specific software providing total/sub-domain scores as well as a rank-based list of ‘weighted environmental barriers’. For each environmental barrier item, the instrument comprises predefined severity ratings, operationalized as points (0–4) that quantify the severity of the problems predicted to arise in the specific case. Thus, the variable accessibility is operationalized as the magnitude of accessibility (P-E fit) problems caused by the case-specific combination of functional limitations/dependence on mobility devices and environmental barriers; higher scores= more accessibility problems. That is, the total accessibility score of the HE represents a function of the individual’s functional limitations, dependence on mobility devices and physical environmental barriers in the home and the closest exterior surroundings. In cases with no functional limitations/dependence on mobility devices, the accessibility score is 0. The theoretical maximum score is 1,832.

##### Perceived aspects of home

Perceived aspects of home comprise four domains defined, operationalized and empirically tested as described in detail by Oswald et al. [29]. *Housing satisfaction* is evaluated with the question “Are you happy with the condition of your home (e.g. structure, roof, ceilings, walls, any dampness etc.)?” The response categories (scored 1–5) are: “No, definitely not”; “No, not to full extent”; “Neither”; “Yes, to some extent”; “Yes, definitely”. *Usability* relates to the degree to which the physical housing environment supports activity performance, and is evaluated with two sub-scales from the Usability in My Home Questionnaire (UIMH): activity aspects (4 items) and physical environmental aspects (6 items). Each item is scored from 1 to 5. Only the end points are defined; these differ between activity aspects (“Not at all suitable”-“Entirely suitable”) and physical environmental aspects (Not at all usable-Fully usable). *Meaning of home* relates to how an individual reacts to and feels about his/her home*,* and is evaluated with the Meaning of Home Questionnaire (MOH). It covers physical (7 items), behavioural (6 items), emotional (10 items) and social aspects (5 items). Each item is scored from 0 (“strongly disagree”) to 10 (“strongly agree”); only the end points are defined. *Housing-related control beliefs* are assessed with the Housing-related Control Beliefs Questionnaire (HCQ). The two included subscales (“Powerful others”; “Chance”) target external control in relation to the home. External control in relation to the home means that “some other person, luck, chance or fate is perceived as explanatory factors for what happens” [29]. Both subscales have 8 items with five response categories (scored 1–5; higher= more external control).

In addition to these four domains, one question targets *neighborhood attachment* (“Are you rooted and feel a strong affinity to your residential area?”) which has four response categories (scored 1 to 4; higher= less rooted) [35]. Another set of questions target *housing adaptations*, regarding the participant’s knowledge about the housing adaptation grant provided by Swedish municipalities as well as whether he/she has been provided such support (Yes/No). In cases where housing adaptations have been accomplished, locations as well as the form for financing the adaptations are to be specified. In addition, the participant is asked about whether he/she thinks that the housing adaptations made have had any impact on daily activities, dependence on help of others and the ability to remain living in the present dwelling. Additional response options are: “small/no effect”, “the situation has worsened” and “other”; to be specified. In relation to the potential impact, several response options may be given.

##### Use and need of assistive devices

Structured questions based on the ISO classification [36] are used to register the use and need of ADs. The interviewer poses the following questions: “Which of the following ADs do you have, and which of them do you use? Are there any ADs which you need but do not have?” This part includes 33 predefined AD items: optical aids (3 items), hearing aids (3 items), mobility devices indoors (6 items) and outdoors (8 items), aids for daily activities (6 items) and other ADs (7 items) such as stair lift and visual door entry system. In addition, the participant can describe the use/need of additional products. A subsequent dichotomous (Yes/No) question specifically targets whether the participant has a security alarm.

*Social support* is explicitly addressed by the following question: “Is there someone around, who could assist you in case you would need some help and support?” If responding yes, the relationship to that person is to be specified.

#### Health aspects

The battery of self-administered questions and questionnaires provided by post is initiated by a self-rating of the current mobility as either Good (“on”); Good, but hyperkinetic; or Bad (“off”), followed by two dichotomous (Yes/No) questions that target fluctuations and dyskinesia. The subsequent self-ratings primarily target: *freezing of gait, walking difficulties, fear of falling, activity avoidance due to a risk of falling, non-motor symptoms, fatigue, general self-efficacy and activities of daily living*. The following questionnaires are included (Table 1): the self-administered version [28] of the Freezing of Gait Questionnaire [37] (FOG-Qsa); the generic Walk-12 (Walk-12G) [24]; Falls Efficacy Scale-International (FES-I) [38]; modified Survey of Activities and Fear of Falling in the Elderly (mSAFFE) [25,39]; the Nonmotor Symptoms Questionnaire (NMSQuest) [26]; the Energy section of the Nottingham Health Profile (NHP-EN) [27]; the General Self-Efficacy Scale (GSE) [40]; and the Parkinson’s Disease Activities of Daily Living Scale (PADLS) [23]. Six additional dichotomous (Yes/No) questions concern *perceived balance problems including near falls*: unsteadiness while walking; dual tasking (“Do you experience balance problems while standing or walking when doing more than one thing at a time, e.g. carrying a tray while walking?”); unsteadiness while turning; fear of falling; activity avoidance due to a risk of falling; and whether the participant has experienced any near falls during the past six months (if yes, the approximate number of times should be specified). A near fall is defined as “a fall initiated but arrested by support from a wall, railing, other person, etc.” [41]. *Dizziness* is screened for by the question: “Have you ever experienced any dizziness in the past year?” If answering ‘yes’, the participant is asked to verify the sensation as: (i) rotational/spinning; (ii) lightheadedness; (iii) other or (iv) don’t know [42]. Several options may be ticked. A single item question (scored 1–5; higher = worse) targets perceived *general health*; inspired by the general health question in the Medical Outcome Study (MOS) 36-item Short-Form health survey [43]. The final question in the self-administered battery concerns whether the participant responded independently or attained assistance.

At the subsequent home visit, initially the participant is asked to verify the time point of the latest intake of anti-PD medications, and to self-rate his/her present state of mobility (see above). *Motor symptoms* are assessed according to the Unified Parkinson’s Disease Rating Scale (UPDRS, part III) (14 items) [44], in which item no. 30 specifically captures the postural response in relation to an external perturbation (scored 0–4). The total score of the UPDRS part III ranges from 0–108 points (higher scores = worse). *Complications of therapy* in the past week (e.g. dyskinesias, fluctuations) are assessed according to the UPDRS part IV (11 items) [44]. The *severity of PD* is assessed according to the Hoehn & Yahr staging scale [45] (ranges from I to V; higher = worse), both for the on and off condition although the latter is based on anamnestic information. *Cognitive functions* are assessed with the Montreal Cognitive Assessment (MoCA) (max. score = 30) [46]. Two *timed tests* target mobility and lower-extremity function, respectively; the Timed Up & Go (three trials) [47,48]) and the Chair-Stand Test (one trial) [49,50]. Details about the standardizations of these two tests are provided by Nilsson et al. [51]. *Pain* is assessed by the dichotomous (Yes/No) question “Are you bothered by pain?” If responding yes, the severity subscale of the brief screening version of the Multidimensional Pain Inventory (Swedish version) is used [52]. It consists of two items (each scored 0–6; higher = more severe pain). Locations of pain are also to be specified.

*Falls* are targeted by several structured questions, applying the European consensus definition of a fall [53]. Initially, the participant is asked a dichotomous (Yes/No) question regarding falls during the past year. If responding yes, a subsequent question concerns whether it happened more than once (Yes/No) including an estimate of how many times. In addition, 13 predefined locations of falls (indoors and outdoors) are to be specified and ranked (1= most common location). One question concerns whether any fall incident required medical care, and the final question targets the occurrence and number of falls during the past six months.

The structured interview during the home visit also targets *psychological wellbeing, coping, life satisfaction and depression*. The used version of the Psychological Wellbeing Questionnaire (PWQ) [54] consists of 19 statements whereas the Coping Pattern Schedule (CPS) has 13 [55]. Both scales have the same response categories (scored 1–5): “strongly disagree”; “disagree”; “neutral”; “agree”; and “strongly agree”. Life satisfaction is evaluated by item 1 (scored 1–6; higher = better) of the Life Satisfaction Questionnaire (LISAT-11) [56]. Depressive symptoms are assessed with the Geriatric Depression Scale (GDS-15) [57]. It consists of 15 dichotomous (Yes/No) items that add to a total score (range 0–15; higher = more depressive symptoms).

*Independence in activities of daily living* (ADL) is assessed by means of observation and interview according to the ADL Staircase [58]. Each of the nine items included (i.e. activities) is rated as independent/partly dependent/dependent. For ADL items rated as “independent”, the interviewer asks a dichotomous (Yes/No) question [59]: “Even if you manage on your own, do you experience any difficulty when performing…?” In addition, *perceived functional independence* (PFI) is addressed by the question “All in all, how would you evaluate your own independence, i.e. in performing activities of daily living?” It is scored from 0 (“completely dependent”) to 10 (“completely independent”); only the endpoints are defined.

*Participation* in life situations is captured by structured questions representing two dimensions; ‘performance-oriented participation’ and ‘togetherness-oriented participation’ [60]. Fifteen questions address the frequency of participating in different social activities. Each of the 15 items has five response categories: “Every day”; “Once/twice a week”; “Once/twice a month”; “Once/twice a year”; and “Nearly never/never”. An additional dichotomous question (Yes/No) addresses membership of any association and if so, whether the individual participates in its organized activities. A similar question specifically targets this in relation to patient associations. Furthermore, the participant is asked to specify three leisure activities (indoors and outdoors, respectively) that he/she likes to do nowadays, whether these activities are performed alone and/or with others, and if there are unmet wishes or barriers in relation to performance. One additional question concerns the frequency of outdoor walks, with five response categories: “Every day”; “Once/twice a week”; “Once/twice a month”; “Nearly never”; and “Never”. Finally, the participant is asked whether he/she has used any activity center services for senior citizens during the latest 12 months.

At the end of the home visit, the participant is asked whether he/she is interested in participating in future follow-ups. After the home visit, the project administrator registers whether any other person participated during the home visit, and if so, in what way and whether it was perceived as influencing the responses of the participant (scored 0–10; higher scores= more impact). The project administrator also registers perceived communication ability (scored 0–10; higher= better) and reliability of responses (scored 1–5; higher= worse).

### Data quality control and software

In order to ensure that the database accurately reflects the data reported in the questionnaires, a proof reading procedure will be carried out. For proof reading 20% of the cases will be randomly selected. Any discrepancies found will be noted on a log sheet and rectified in the database. Thereafter an error rate will be calculated that should not exceed 0.5%. If the error rate is higher the proof-reading procedure will be extended to cover 100% of the cases. In addition to the proof-reading procedure a validation of the data will be performed by checking for ranges, logical consistency and completeness. Missing or unclear data will undergo a data cleaning process, where Data Clarification Forms will be used. Any changes applied to data in the database during data cleaning will be noted on a log sheet. After completion of data cleaning the database will be locked.

For the data recording and data cleaning processes appropriate validated software will be used (SAS Version 9.3; IBM SPSS Statistics version 20; Housing Enabler 2.0).

### Statistical analysis plan

Different data analyse approaches will be applied, depending on the specific research questions. Depending on the data level for the variables under study, parametric as well as non-parametric statistics will be used. Since measurement properties such as validity and reliability are sample dependent [61,62], detailed information regarding measurement properties will be provided in forthcoming studies based on the baseline data collected. Such analyses will target data quality (e.g. percentage of missing item responses), basic scaling assumptions (e.g. floor and ceiling effects) and reliability (Cronbach’s alpha) [62].

In order to determine how e.g. accessibility problems, usability of the home in relation to activities, and participation restrictions are related and evolve over time, the analyses of contributing or predictive factors require multivariate regression analysis. It is then recommended to have at least 100 participants [63], and the sample size should be five to ten times the number of variables [64]. Potential relationships between home and health aspects will be investigated by means of correlation analyses and Structural Equation Modelling Techniques (SEM).

## Discussion

The project presented in this study protocol paper enters unbroken ground within PD-research by addressing home and health dynamics, with attention to PD-specific symptomatology such as balance problems, falls and gait problems. The means for tackling these gaps of knowledge is to merge different research traditions and perspectives, i.e. neurology, gerontology, health science and rehabilitation, using state-of-the art methodology from different fields of inquiry. Since we did not posit any age span as an inclusion criterion, the project also acknowledges the criticism that older people are often excluded from PD-research [3].

The integration of theory and methodology from several research fields in a complex longitudinal research project requires careful planning. Through the already published explorative studies we gained important experiences for the problem and research questions definition as well as for the data collection and the forthcoming analyses. In addition, recent studies based on the ENABLE-AGE database generated new knowledge e.g. on relocation along the process of ageing [65,66]. This is of relevance also for PD-research since people with PD are more likely to move to assisted living facilities and at an earlier age [1]. That is, understanding objective as well as perceived aspects of home in this target group might be even more important than among older people in general. Furthermore, we are in a strong position to deliver novel comparative studies, comparing people with a PD-diagnose with matched controls selected from the ENABLE-AGE database or other databases available (PI: S. Iwarsson). For such comparative studies, we will use a design with several controls per PD participant and matching criteria such as sex, age and housing type, as applied in the preparatory studies [21,22].

The knowledge generated by the project presented in this paper will be of value for the planning of health care (including rehabilitation and the provision of ADs) and social services, and for the provision of individual housing adaptations and accessible housing options that meet the specific needs of people diagnosed with PD. As yet, such interventions and societal planning are seldom based on scientific evidence, but have the potential to support active and healthy ageing also for this target group. The results of this project will contribute to the much needed knowledge base for the development of evidence-based interventions and foresighted societal planning promoting activity, participation and quality of life for those ageing with PD.

## Competing interests

The first author (MHN) declares no competing interests. The second author (SI) is the shared copyright holder and owner of the Housing Enabler (HE) instrument and software, provided as commercial products (see http://www.enabler.nu).

## Authors’ contributions

The authors (MHN and SI) conceived the idea for the project and jointly designed it. MHN drafted the initial manuscript for submission to *BMC* Neurology. The two authors collaborated in the development and finalization of the manuscript and both approved the final version.

## Pre-publication history

The pre-publication history for this paper can be accessed here:

http://www.biomedcentral.com/1471-2377/13/142/prepub
